# S6K1 controls adiponectin expression by inducing a transcriptional switch: BMAL1-to-EZH2

**DOI:** 10.1038/s12276-022-00747-7

**Published:** 2022-03-25

**Authors:** Sang Ah Yi, Ye Ji Jeon, Min Gyu Lee, Ki Hong Nam, Sora Ann, Jaecheol Lee, Jeung-Whan Han

**Affiliations:** 1 School of Pharmacy, Sungkyunkwan University, Suwon, 16419 Republic of Korea; 2 Biomedical Institute for Convergence at SKKU (BICS), Sungkyunkwan University, Suwon, 16419 Korea; 3 Imnewrun Biosciences, Inc, Suwon, 16419 Korea

**Keywords:** Phosphorylation, Gene silencing, Transcriptional regulatory elements

## Abstract

Adiponectin (encoded by *Adipoq*), a fat-derived hormone, alleviates risk factors associated with metabolic disorders. Although many transcription factors are known to control adiponectin expression, the mechanism underlying its fluctuation with regard to metabolic status remains unclear. Here, we show that ribosomal protein S6 kinase 1 (S6K1) controls adiponectin expression by inducing a transcriptional switch between two transcriptional machineries, BMAL1 and EZH2. Active S6K1 induced a suppressive histone code cascade, H2BS36p-EZH2-H3K27me3, leading to suppression of adiponectin expression. Moreover, active S6K1 phosphorylated BMAL1, an important transcription factor regulating the circadian clock system, at serine 42, which led to its dissociation from the *Adipoq* promoter region. This response resulted in EZH2 recruitment and subsequent H3K27me3 modification of the *Adipoq* promoter. Upon fasting, inactivation of S6K1 induced the opposite transcriptional switch, EZH2-to-BMAL1, promoting adiponectin expression. Consistently, S6K1-depleted mice exhibited lower H3K27me3 levels and elevated adiponectin expression. These findings identify a novel epigenetic switch system by which S6K1 controls the production of adiponectin, which displays beneficial effects on metabolism.

## Endocrinology: Revealing the regulation of a fat-derived hormone

Insights into the regulatory machinery controlling production of the hormone adiponectin could guide development of treatments for various metabolic disorders. Fat cells secrete adiponectin in response to nutrient deprivation as well as circadian cues, and evidence suggests this hormone is protective against diabetes, obesity, and hypertension. Sang Ah Yi from Sungkyunkwan University, Suwon, South Korea and colleagues have determined that a protein called S6K1 plays a central role in governing adiponectin production. They show that S6K1 maintains the adiponectin gene in an inactive state via a two-pronged inhibitory mechanism. S6K1 interacts closely with both the cellular circadian clock and other epigenetic pathways, and is inactivated by fasting conditions, allowing adiponectin synthesis to proceed. These regulatory pathways could offer a useful target for therapeutic efforts aimed at boosting adiponectin production.

## Introduction

Transcriptional switching is a principal regulatory mechanism for cell fate determination, especially during cell cycle control and differentiation. In particular, polycomb complex switching during the neuronal differentiation system is one of the best-known epigenetic regulatory systems. During differentiation, transcription factors of lineage-specific genes are activated, and H3K27me3 is eliminated by the appropriate signaling pathway^1^. An epigenetic switch controls both positive and negative effectors of gene transcription with a complex interaction, in which the switch state is determined by the relative concentrations of both effectors, the selective affinity for the target binding site, and the location within the nucleus of the cell^2^. Thus, defining the precise interplay between epigenetic factors and transcription factors as gene regulators is required to control specific gene regulatory networks.

Adiponectin, produced and secreted from adipose tissues, is inversely associated with multiple metabolic symptoms, such as insulin resistance, hypertension, and dyslipidemia^3^. According to the ‘adiponectin hypothesis’, hypoadiponectinemia is a hallmark of obesity and might cause obesity-related diseases such as cardiovascular diseases and type 2 diabetes^4^. Thus, increasing adiponectin production is a useful strategy to ameliorate obesity-mediated metabolic diseases. To date, adiponectin expression has been shown to be regulated by adipogenic transcription factors such as peroxisome proliferator-activated receptor γ (PPARγ), CCAAT/enhancer-binding protein (C/EBP), and sterol regulatory element-binding transcription factor 1 (SREBPF1)^5^. However, the exact signaling pathway, epigenetic modifications and transcriptional machinery responsible for the dynamic fluctuation of adiponectin expression are still unclear.

S6K1, a key downstream effector of the mammalian target of rapamycin (mTOR) signaling pathway, plays a critical role in nucleotide, lipid, and protein synthesis. S6K1 was recently shown to rhythmically phosphorylate the key circadian transcription factor BMAL1 in the cytosol^6^. S6K1-mediated phospho-BMAL1 is a critical factor for stimulating protein synthesis in a circadian manner. The majority of S6K1 studies have focused on cytosolic roles, but accumulated evidence has shown the localization of S6K1 in the nucleus^7,8^. Our previous study demonstrated that nuclear S6K1 induces early adipogenesis by inhibiting *Wnt* gene expression through H2BS36 phosphorylation, recruiting EZH2, and subsequent H3K27me3 modification, suggesting the epigenetic role of S6K1 in the nucleus^9^. Here, we show the role of S6K1 in epigenetic switching that controls adiponectin gene expression. In addition to activating the cascade of epigenetic suppressors consisting of H2BS36p, EZH2, and H3K27me3, S6K1 prevents BMAL1 from acting as a positive effector of adiponectin expression by phosphorylating nuclear BMAL1. Collectively, the S6K1-mediated epigenetic switch is responsible for fine control of adiponectin expression.

## Materials and methods

### Cell line and differentiation

The 3T3-L1 cell line was cultured in high-glucose Dulbecco’s modified Eagle’s medium (DMEM; Welgene, Seoul, Korea, Cat. No. LM001-05) supplemented with 10% bovine calf serum (BCS; Welgene, Cat. No. S103-01) and 1% penicillin/streptomycin (P/S; Welgene, Cat. No. LS202-02) at 37 °C in a humidified 5% CO_2_ incubator. For adipocyte differentiation, 80% confluent 3T3-L1 cells were incubated for 2 days in high-glucose DMEM with 10% fetal bovine serum (FBS; Welgene, Cat. No. S001-07), 1% P/S, 0.5 mM 3-isobutyl-1-methylxanthine (IBMX; Sigma, St.Louis, MO, USA, Cat. No. I5879), 1 µM dexamethasone (Sigma, Cat. No. D1756), and 10 µg/mL insulin (Sigma, Cat. No. I9278). Then, 3T3-L1 cells were incubated for 6–8 days in high-glucose DMEM with 10% FBS, 1% P/S, and 10 µg/mL insulin. The medium was changed every other day.

### Mice

S6K1‐deficient C57BL/6 mice were a generous gift from George Thomas and Sara C. Kozma (IDIBELL and University of Cincinnati), and C57BL/6 mice and CF-1 mice were purchased from Daehan BioLink. The Sungkyunkwan University Institutional Animal Care and Use Committee (SKKUIACUC) approved the experimental procedures and care of the animals. All procedures performed in this study involving animals were in accordance with the guidelines of the SKKUIACUC. WT and S6K1‐deficient mice were housed in standard plastic cages in a controlled environment at a temperature of 22 ± 2 °C, humidity of 50 ± 5%, and 12:12 h light-dark cycle with 10–18 air changes per hour. Mice were supplied with a basal diet and sterilized water without any restrictions during the experiment. For the fasting experiments, C57BL/6 mice were fasted at the onset of the dark cycle, and adipose tissue was isolated by sacrificing the mice 24 h after fasting. Mice used for brown adipose tissue, inguinal white adipose tissue and epididymal white adipose tissue were between 8 and 10 weeks old.

### Antibodies and constructs

The primary antibodies used in this study are listed in Supplementary Table 1. The DNA constructs used in this study were pCDNA-EGFP-H2B, pCDNA-EGFP-H2BS36A, pCDNA-EGFP-H2BS36D, and pRK5-myc-S6K1-CA, which were previously described^9^. pCMV6-FLAG-BMAL1 was purchased from Origene (Rockville, MD, USA, Cat. No. MR209553). The mutant constructs for BMAL1 were generated using site-directed mutagenesis (pCMV6-FLAG-BMAL1-S42A).

### Inhibition of S6K1

First, 3T3-L1 adipocytes were treated with rapamycin (Calbiochem, San Diego, CA, USA, Cat. No. 553210) or PF-4708671 (Tocris, Bristol, UK, Cat. No. 4032) to inhibit S6K1 activity. For S6K1 knockdown, fully differentiated 3T3-L1 cells were transfected with siRNA targeting S6K1 using Lipofectamine 2000 (Invitrogen, Waltham, MA, USA, Cat. No. 11668019) according to the manufacturer’s protocol. The sequences of the siRNAs targeting S6K1 were as follows: #03 forward, 5′-GGACCAGCCAGAAGAUGCAGGCUCU-3’; #03 reverse, 5’-AGAGCCUGCAUCUUCUGGCUGGUCC-3′; #04 forward, 5′-CACCCUUUCAUUGUGGACCUGAUUU-3′ and #04 reverse, 5′-AAAUCAGGUCCACAAUGAAAGGGUG-3′.

### Suppression of EZH2

First, 3T3-L1 adipocytes were transfected with the pLKO.1 vector encoding EZH2 shRNA using Lipofectamine 2000 (Invitrogen, Cat. No. 11668019) according to the manufacturer’s protocol. The shRNA sequences targeting EZH2 were as follows: #05, CCGGACTTGCCCACCTCGGAAATTTCTCGAGAAATTTCCGAGGTGGGCAAGTTTTTTG; #06, CCGGGCACAAGTCATCCCGTTAAAGCTCGAGCTTTAACGGGATGACTTGTGCTTTTTG; #39, CCGGGCGTATAAAGACACCACCTAACTCGAGTTAGGTGGTGTCTTTATACGCTTTTTG; #43, CCGGGCTGACCATTGGGACAGTAAACTCGAGTTTACTGTCCCAATGGTCAGCTTTTTG; #66, CCGGAGTCGCCTCGGTGCCTATAATCTCGAGATTATAGGCACCGAGGCGACTTTTTTG.

### Suppression of BMAL1

We transfected 3T3-L1 adipocytes with siRNA targeting BMAL1 using Lipofectamine 2000 (Invitrogen, Cat. No. 11668019) according to the manufacturer’s protocol. The sequences of the siRNAs targeting BMAL1 were as follows: forward, 5′- CCACCAACCCAUACACAGAAGCAAA-3′ and reverse, 5′- UUUGCUUCUGUGUAUGGGUUGGUGG-3′.

### Protein extraction and immunoblotting

Total protein lysates were extracted using PRO-PREP reagent (Intron, Seongnam, Korea, Cat. No. 17081). Lysates were homogenized by ultrasonic homogenizers for 5 sec at 12% amplitude, incubated in ice for 10 min, and centrifuged at 13,000 rpm at 4 °C for 20 min. The protein concentration of the supernatants was measured using Bradford dye on a spectrophotometer. Then, 15–30 µg of protein was used for immunoblotting. The protein samples were subjected to homemade sodium dodecyl sulfate-polyacrylamide gel electrophoresis (Bio-Rad, Hercules, CA, USA). Subsequently, the proteins were transferred onto polyvinylidene difluoride membranes (Millipore, Burlington, MA, USA) using a semidry transfer system (Bio-Rad). The blocked membranes treated with 5% skim milk in TBST (50 mM Tris pH 8.0, 150 mM NaCl, 0.1% Tween-20) were incubated with the indicated primary antibodies at 4 °C for 16 h by rocking. The membranes were incubated with horseradish peroxidase-conjugated secondary antibodies in 5% skim milk in TBST at room temperature for 1 h. Signals were detected using a chemiluminescence reagent (Abclon, Guro, Korea, Cat No. ABC-3001) on AGFA 100 NIF X-ray film (Agfa-Gevaert, Mortsel, Belgium). The signal of the immunoblot band was quantified using ImageJ software.

### Reverse transcription-real time polymerase chain reaction

Total RNA was extracted from 3T3-L1 adipocytes or isolated tissues and mouse tissues using Easy-Blue reagent (Intron, Cat. No. 17061). In all, 1 μg of RNA was reverse transcribed into cDNA using the Maxime RT PreMix kit (Intron, Cat. No. 25081). qPCR was performed using KAPATM SYBR FAST qPCR (Kapa Biosystems, Wilmington, MA, USA, Cat. No. KK4601) with a CFX96TM real-time PCR detector (Bio-Rad). The relative mRNA levels were normalized to the *β-actin* mRNA levels for each target gene. The qPCR primer sequences used in this study are listed in Supplementary Table 2.

### Cytoplasmic and nuclear fractionation

The cells were washed using cold phosphate-buffered saline (PBS, pH 7.2) and suspended in buffer A (10 mM HEPES (pH 7.9) containing 1.5 mM MgCl_2_, 10 mM KCl, 1 mM EDTA, 1 mM dithiothreitol (DTT), 0.5 µg/mL leupeptin, 1 mM phenylmethylsulfonylfluoride (PMSF), 1 μM pepstatin A, and 0.05% NP-40). The cell lysates were incubated on ice for 10 min. The cytoplasmic extracts were separated by centrifugation at 3000 rpm at 4 °C for 10 min. The supernatants were used as cytoplasmic extracts. The remaining pellet was resuspended in buffer B (20 mM HEPES (pH 7.9) containing 1.5 mM MgCl_2_, 420 mM KCl, 25% glycerol, 0.2 mM EDTA, 1 mM DTT, 0.5 μg/mL leupeptin, 1 mM PMSF, and 1 μM pepstatin A) and incubated on ice for 30 min. The nuclear extracts were separated by centrifugation at 13,000 rpm and 4 °C for 20 min. The cytoplasmic and nuclear extracts were analyzed by immunoblotting.

### Immunoprecipitation

Fully differentiated 3T3-L1 cells were lysed with immunoprecipitation (IP) lysis buffer (40 mM HEPES (pH 7.4) containing 120 mM NaCl, 1 mM EDTA, 50 mM NaF, 1.5 mM Na_3_VO_4_, 10 mM β-glycerophosphate, 0.3% CHAPSO, and protease inhibitors). The lysates were centrifuged for 20 min at 13,000 rpm at 4 °C. The indicated antibodies were incubated with the supernatants at 4 °C for 16 h by rotation. The mixtures of protein and antibodies were incubated with SureBeadsTM Protein A magnetic beads (Bio-Rad, Cat. No. 161-4013) at 4 °C for 1 h. The complexes of beads, antibodies and target proteins were spun down for 1 min at 2,000 rpm at room temperature and washed with IP wash buffer (IP lysis buffer without CHAPSO) three times. The proteins were eluted by boiling for 5 min in Laemmli sample buffer (Bio-Rad, Cat. No. 1610737). Input protein and immunoprecipitated extracts were analyzed by immunoblotting.

### Chromatin immunoprecipitation

The chromatin immunoprecipitation assay was performed using a Zymo-Spin ChIP kit (Zymo Research, Irvine, CA, USA, Cat. No. D5210) as previous described^10^. Chromatin was crosslinked with 1% formaldehyde for 10 min at room temperature. The crosslinking reaction was stopped at a final concentration of 0.125 M glycine. The prepared nuclei were mechanically sheared using the Bioruptor Pico sonication system (Diagenode, Liege, Belgium) at 4 °C in 30 s on/off for 25 cycles. A small portion of the chromatin solution was reserved as input DNA. The chromatin samples were incubated with the indicated antibodies by rotating for 16 h at 4 °C. SureBeads Protein A/G magnetic beads (Bio-Rad, Cat. No. 161-4013) were incubated at 4 °C for 1 h by rotation to elute the antibody-chromatin complexes. The bead-antibody-chromatin complex was washed three times with chromatin wash buffer (Zymo Research, Cat. No. D5210). The eluted chromatin was decrosslinked with 5 M NaCl, and then, the chromatin proteins were degraded with proteinase K (genDEPOT, Katy, TX, USA, Cat. No. P2170). ChIP DNA was purified using a QIAquick Spin column (Qiagen, Hilden, Germany, Cat. No. 28115). The ChIP DNA and input were analyzed via qPCR using primer pairs specific to the target gene promoters. The primer sequences are listed in Supplementary Table 2.

### Statistical analysis

Statistical significance was determined using Student’s *t* test (two-tailed) and assessed based on the P value. The results with *P < 0.05, **P < 0.01 and ***P < 0.001 were considered statistically significant.

## Results

### S6K1 regulates adiponectin expression by directly binding to its promoter

**Fig. 1:**
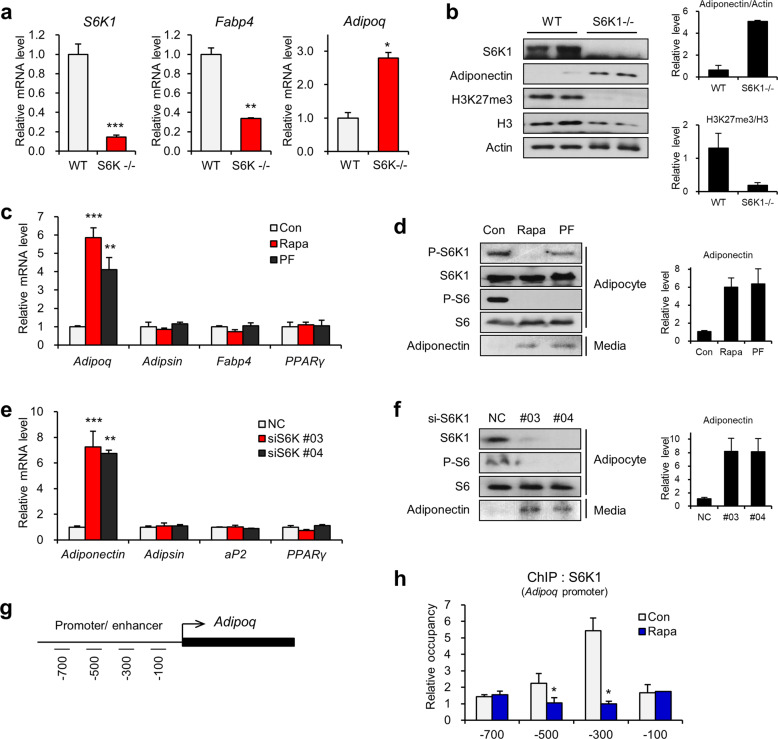
S6K1 negatively regulates adiponectin expression by directly binding to the promoter. **a** The mRNA levels of *S6k1*, *Fabp4*, and *Adipoq* in epididymal white adipose tissue obtained from the wild-type or S6K1^-/-^ mice. **b** Immunoblot analysis of epididymal white adipose tissue obtained from the wild-type or S6K1^−/−^ mice. **c** The mRNA levels of *Adipoq*, *Adipsin*, *Fabp4*, and *Pparγ* in the 3T3-L1 adipocytes treated with rapamycin (25 nM) or PF-4708671 (10 μM) for 3 h. **d** Immunoblot analysis of whole-cell lysates and secreted adiponectin protein levels in the culture medium of the 3T3-L1 adipocytes treated with rapamycin (25 nM) or PF-4708671 (10 μM) for 3 h. **e** The mRNA levels of *Adipoq*, *Adipsin*, *Fabp4*, and *Pparγ* in the 3T3-L1 adipocytes transfected with S6K1 siRNA (#03 or #04). **f** Immunoblot analysis of whole-cell lysates and secreted adiponectin protein levels in the culture medium of the 3T3-L1 adipocytes transfected with S6K1 siRNA (#03 or #04) or negative control siRNA (NC). **g** Schematic representation of the promoter/enhancer region of *Adipoq*. Underlines show the four primer locations for ChIP-qPCR. **h** ChIP-qPCR analysis using S6K1 antibody on the promoter regions (−700, −500, −300, and −100 bp) of *Adipoq* in the 3T3-L1 adipocytes treated with rapamycin (25 nM) for 3 h. Data are represented as the mean ± SEM for *n* = 3. *P < 0.05; **P < 0.01; ***P < 0.001.

To identify the relationship between S6K1 and adiponectin expression, we initially examined the expression of adiponectin in epididymal white adipose tissue of S6K1-deficient mice. While the mRNA levels of *Adipoq* were elevated, those of *S6k1* and *Fabp4* were decreased in the S6K1-deficient mice (S6K^-/-^) compared to the wild-type (WT) mice (Fig. 1a). Notably, the global levels of H3K27me3 showed an opposite pattern compared to the levels of adiponectin in white adipose tissue (Fig. 1b). These results suggest that adiponectin expression is regulated by S6K1-mediated H3K27me3, a repressive epigenetic marker in white adipocytes.

Since we previously showed that the nuclear localization of S6K1 is essential for inducing repressive epigenetic modifications during early adipogenesis^9^, we performed immunocytochemistry to visualize the spatial localization of S6K1 and H3K27me3 in mature adipocytes. Interestingly, S6K1 occupied distinct territories forming small puncta in the nucleus in undifferentiated adipose precursor cells (Supplementary Fig. 1, top), whereas the S6K1 puncta were diffused in the nucleoplasm of mature adipocytes (Supplementary Fig. 1, middle). In addition, H3K27me3 was detected in specific interior regions of the nucleus in preadipocytes, while the H3K27me3 signal was enriched at the periphery of the nucleus in differentiated adipocytes (Supplementary Fig. 1, top and middle). Upon rapamycin treatment, S6K1 diffused to the cytoplasm, and H3K27me3 moved further toward the nuclear periphery from the central region of the nucleus (Supplementary Fig. 1, bottom), indicating that the position shift of H3K27me3 within the nucleus depends on the activity of S6K1.

We next investigated whether S6K1 signal transduction is a key regulatory mechanism for adiponectin expression in differentiated adipocytes. Treatment of adipocytes with the mTOR inhibitor rapamycin or the selective S6K1 inhibitor PF-4708671 increased the mRNA expression level of adiponectin (Fig. 1c) and the protein level of adiponectin secreted into the culture medium (Fig. 1d). However, there was no significant change in the expression of other adipocyte marker genes, such as *Adipsin*, *Fabp4*, or *Pparγ*. Consistent with this finding, depletion of S6K1 with siRNA increased both the adiponectin mRNA and protein levels, as shown by increased secretion of adiponectin in the culture medium (Fig. 1e, f). Collectively, the data indicate that S6K1 is a major regulator of adiponectin expression.

To test whether S6K1 is a direct regulator of adiponectin expression, we performed chromatin immunoprecipitation (ChIP) analysis to corroborate the binding of S6K1 to the *Adipoq* promoter in fully differentiated adipocytes. ChIP-qPCR analysis showed that S6K1 bound to the −500 bp and −300 bp regions of the *Adipoq* promoter, and this binding was strongly ablated by rapamycin treatment (Fig. 1g, h). Taken together, these findings suggest that S6K1 directly binds to the adiponectin promoter, thereby playing a role in the transcriptional regulation of adiponectin.

### S6K1-dependent histone crosstalk suppresses adiponectin expression

**Fig. 2:**
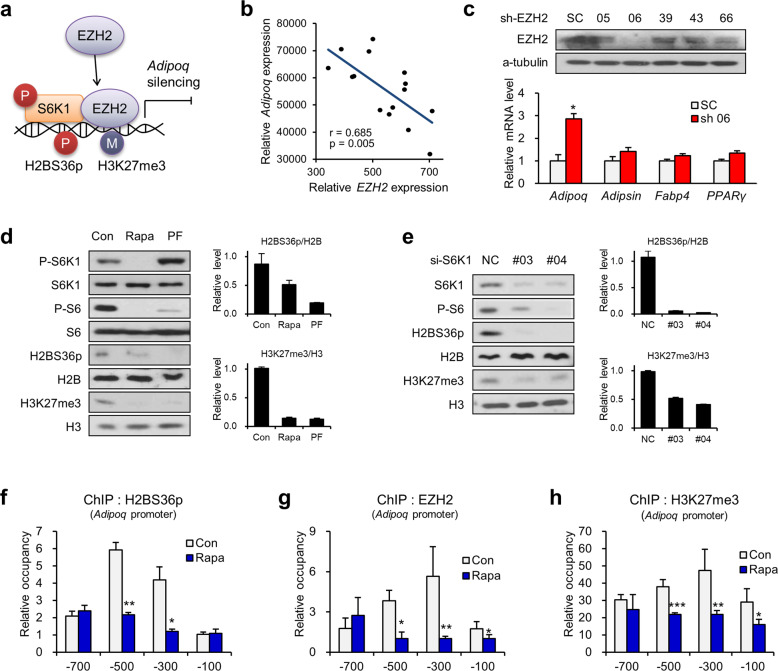
S6K1-mediated H2BS36 phosphorylation and H3K27 trimethylation suppress adiponectin expression. **a** Schematic diagram of the mechanisms of histone crosstalk mediated by S6K1. **b** Pearson’s correlation analysis of *Adipoq* and Ezh2 expression in epididymal fat tissues from rats (GDS2946). **c** Immunoblot analysis of EZH2 after transduction with lentivirus targeting EZH2 (EZH2 shRNA 05, 06, 39, 43, or 66) or control shRNA (SC). The mRNA levels of *Adipoq*, *Adipsin*, *Fabp4*, and *Pparγ* after lentiviral transduction with shEZH2 06 or control shRNA. **d** Immunoblot analysis of the 3T3-L1 adipocytes treated with rapamycin (25 nM) or PF-470867 (10 μM) for 3 h. **e** Immunoblot analysis of the 3T3-L1 adipocytes transfected with S6K1 siRNA (#03 or #04) or negative control siRNA (NC). **f**–**h** ChIP-qPCR analysis using H2BS36p (**f**), EZH2 (**g**), and H3K27me3 (**h**) antibodies of the promoter regions (−700, −500, −300, and −100 bp) of *Adipoq* in the 3T3-L1 adipocytes treated with rapamycin (25 nM) for 3 h. Data are represented as the mean ± SEM for *n* = 3. *P < 0.05; **P < 0.01; ***P < 0.001.

S6K1 has previously been shown to induce H3K27me3 on the promoter region of *Wnt* genes through direct phosphorylation of H2B at serine 36, leading to transcriptional inactivation during early adipogenesis^9^. Thus, we next examined whether the phosphorylation of H2B at serine 36 by S6K1 could be the key regulatory mechanism of adiponectin expression through sequential recruitment of EZH2 and induction of H3K27me3 (Fig. 2a). Low adiponectin expression was observed in brown adipose tissues, whereas adiponectin was rich in epididymal and inguinal white adipose tissue (Supplementary Fig. 2a). The global level of H3K27me3 in brown adipose tissues was elevated compared to that in white adipose tissues (Supplementary Fig. 2a). The mRNA level of EZH2 was low in epididymal and inguinal white adipose tissues obtained from wild-type mice showing high adiponectin expression and vice versa in individuals showing low adiponectin expression (Supplementary Fig. 2b, c). Consistent with this observation, analysis of public data from GEO profiles (GDS2946) showed a negative correlation between the expression levels of EZH2 and adiponectin (Fig. 2b). Depletion of EZH2 in adipocytes enhanced the transcription of *Adipoq*, whereas other adipocyte markers, such as *Adipsin*, *Fabp4*, and *Pparγ*, showed no significant change (Fig. 2c). These data indicate that EZH2 might be an inhibitory regulator of adiponectin expression.

Given that site-specific histone modifications play an important role in gene expression, we screened histone modifications associated with S6K1 in fully differentiated white adipocytes in the absence or presence of the selective S6K1 inhibitor PF-4708671 or the mTOR inhibitor rapamycin. The global levels of H2BS36p and H3K27me3 were diminished upon treatment with the S6K1 inhibitor (Fig. 2d). Similar results were obtained in the S6K1-depleted adipocytes (Fig. 2e). Next, we assessed whether global histone modification changes induced by S6K1 affect the transcription of adiponectin using ChIP analysis of the *Adipoq* promoter with specific histone modifications. The recruitment of S6K1 to *Adipoq* promoter regions (Fig. 1h) was accompanied by increased H2BS36p, and both of these responses were abrogated by rapamycin treatment (Fig. 2f). In addition, we found that the recruitment of EZH2 is correlated with H3K27me3 enrichment at the −500 bp to −100 bp region of the *Adipoq* promoter, and this process was decreased by rapamycin treatment (Fig. 2g, h). Together, the results indicate that S6K1-mediated H2BS36p leads to enrichment of EZH2 and H3K27me3 on the *Adipoq* promoter, thereby suppressing adiponectin expression in mature adipocytes.

**Fig. 3:**
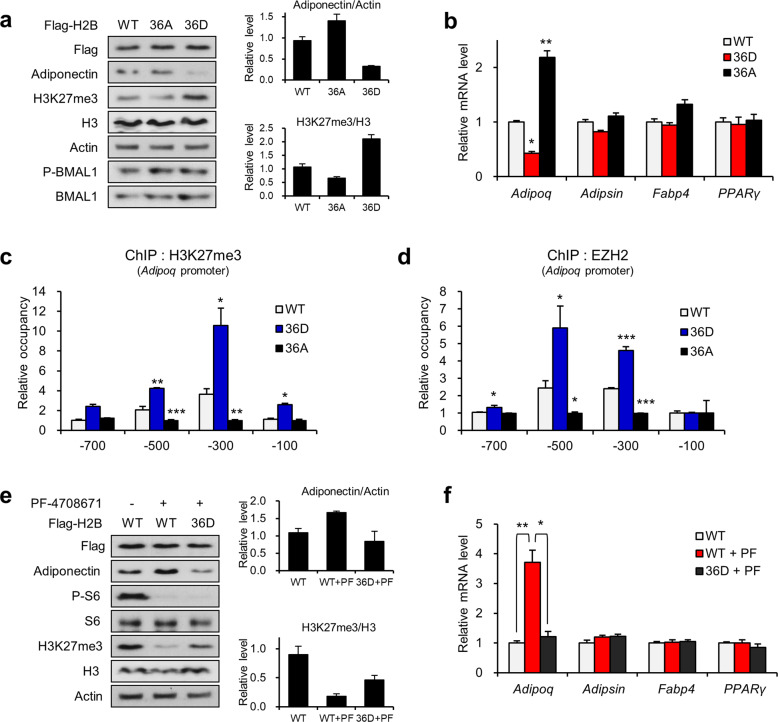
S6K1-mediated H2B serine 36 phosphorylation is required for suppression of adiponectin by EZH2. **a** Immunoblot analysis of the 3T3-L1 adipocytes overexpressing wild-type H2B (WT) H2BS36D (36D) or H2BS36A (36A). **b** The mRNA levels of *Adipoq*, *Adipsin*, *Fabp4*, and *Pparγ* in the 3T3-L1 adipocytes overexpressing wild-type H2B (WT), H2BS36D (36D), or H2BS36A (36A). **c, d** ChIP-qPCR analysis using H3K27me3 (**c**) and EZH2 (**d**) antibodies of the promoter regions (−700, −500, −300, and −100 bp) of *Adipoq* in the 3T3-L1 adipocytes overexpressing wild-type H2B (WT), H2BS36D (36D), or H2BS36A (36A). **e** Immunoblot analysis of the 3T3-L1 adipocytes overexpressing wild-type H2B (WT) or H2BS36D (S36D) treated with PF-4708671 (10 μM) for 3 h. **f** The mRNA levels of *Adipoq*, *Adipsin*, *Fabp4*, and *Pparγ* in the 3T3-L1 adipocytes overexpressing H2B-WT or H2BS36D treated with PF-4708671 (10 μM) for 3 h. Data are represented as the mean ± SEM for *n* = 3. *P < 0.05; **P < 0.01.

We next addressed whether H2B phosphorylation by S6K1 is a major axis component that controls adiponectin expression. The ectopic expression of the phosphomimetic H2B mutant H2BS36D resulted in upregulation of H3K27me3 levels and downregulation of adiponectin expression, while the phospho-resistant H2B mutant H2BS36A showed the opposite effects (Fig. 3a). Consistently, the ectopic expression of H2BS36D specifically downregulated the mRNA level of adiponectin but had no effect on that of *Pparγ, Adipsin*, and *Fabp4* in fully differentiated white adipocytes (Fig. 3b). Consistently, the enrichment of H3K27me3 and EZH2 on the *Adipoq* promoter was increased by ectopic expression of H2BS36D but decreased by H2BS36A (Fig. 3c, d). In accordance with these data, the white adipocytes transfected with H2BS36D showed resistance to the S6K1 inhibitor-mediated increase in adiponectin levels (Fig. 3e, f). Likewise, the S6K1 inhibitor-induced reduction in global H3K27me3 levels was restored upon H2BS36D expression. Thus, S6K1-mediated suppression of adiponectin was caused by a cascade of histone modifications, H2BS36p and H3K27me3.

### S6K1-dependent BMAL1 phosphorylation downregulates Adipoq expression

The *Adipoq* promoter contains E-box-like sequences that can be occupied by helix-loop-helix (HLH) transcription factors such as BMAL1 (Brain and Muscle ARNT-Like 1). BMAL1 forms a heterodimer with CLOCK and acts as a transcription factor for the *Cry* and *Per* genes that determine circadian rhythms^11^. However, the precise role of BMAL1 in the transcriptional regulatory mechanism of adiponectin remains unclear^12^. A previous study showed that BMAL1, which is phosphorylated by S6K1 on serine 42, rhythmically interacts with translational machinery in the cytoplasm and promotes protein synthesis^6^.

**Fig. 4:**
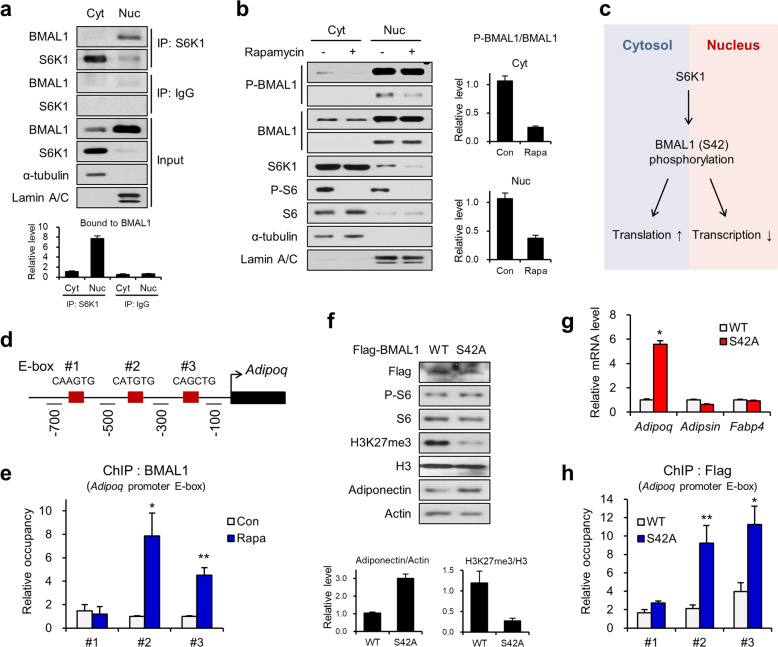
S6K1-mediated BMAL1 phosphorylation downregulates adiponectin expression. **a** Immunoblot analysis of immunoprecipitates (IP) using S6K1 or IgG antibody and whole-cell lysates (input) from separated cytosolic and nuclear extracts of 3T3-L1 adipocytes. Lamin A/C was used as a nuclear marker, and α-tubulin was used as a cytoplasmic marker. **b** Immunoblot analysis of separated cytosolic and nuclear extracts of the 3T3-L1 adipocytes treated with rapamycin (25 nM) for 3 h. **c** Schematic representation of the role of BMAL1 phosphorylated by S6K1 in the cytosol and nucleus. **d** Schematic representation of the three E-box regions of *Adipoq*. E-box #1, #2, and #3 primer-binding sites for ChIP-qPCR. **e** ChIP-qPCR analysis using a BMAL1 antibody on E-box #1, #2, and #3 of *Adipoq* in the 3T3-L1 adipocytes treated with rapamycin (25 nM) for 3 h. **f** Immunoblot analysis of the 3T3-L1 adipocytes overexpressing Flag-BMAL1-wild-type (WT) or Flag-BMAL1-S42A (S42A). **g** The mRNA levels of *Adipoq*, *Adipsin*, and *Fabp4* in the 3T3-L1 adipocytes overexpressing Flag-BMAL1-WT or Flag-BMAL1-S42A. **h** ChIP-qPCR analysis using Flag antibody on E-box #1, #2, and #3 of *Adipoq* in the 3T3-L1 adipocytes overexpressing Flag-BMAL1-WT or Flag-BMAL1-S42A. Data are represented as the mean ± SEM for *n* = 3. *P < 0.05; **P < 0.01; ***P < 0.001.

However, BMAL1 was predominantly detected in the nucleus rather than in the cytoplasm of fully differentiated adipocytes (Fig. 4a). Moreover, the interaction between S6K1 and BMAL1 was observed in the nuclei (Fig. 4a). S42 phosphorylation of BMAL1 was ablated by rapamycin treatment in both the cytoplasm and the nucleus (Fig. 4b), suggesting S6K1-mediated phosphorylation of BMAL1 in the nucleus. Similarly, the level of phosphorylated BMAL1 was also decreased upon S6K1 depletion (Supplementary Fig. 3a). These data indicate that BMAL1 phosphorylated by S6K1 functions in the nucleus in addition to playing an established role in the cytosol^6^ (Fig. 4c).

Chromatin fractionation data showed that most BMAL1, as well as S6K1 and EZH2, were found in the insoluble chromatin-bound protein fraction in both 3T3-L1 preadipocytes and mature adipocytes (Supplementary Fig. 3b). To elucidate the transcriptional function of BMAL1 in adiponectin expression, we evaluated whether BMAL1 directly binds to the three E-box regions of the *Adipoq* promoter (Fig. 4d). The enrichment of BMAL1 on the *Adipoq* promoter was increased by rapamycin treatment, indicating the negative function of S6K1 in the recruitment of BMAL1 to the *Adipoq* promoter (Fig. 4e). The phospho-resistant BMAL1-S42A mutant, which still possesses transcription factor activity, abolished global H3K27me3 modification (Fig. 4f) and thus induced adiponectin expression (Fig. 4f, g). Furthermore, the occupancy of BMAL1-S42A on the *Adipoq* E-box was higher than that of wild-type BMAL1 (Fig. 4h). These data indicate that the unphosphorylated form of BMAL1 is required for its recruitment to the *Adipoq* promoter region to induce adiponectin transcription and that S6K1 acts as a negative regulator of BMAL1 transcriptional activity by phosphorylating S42 of BMAL1.

**Fig. 5:**
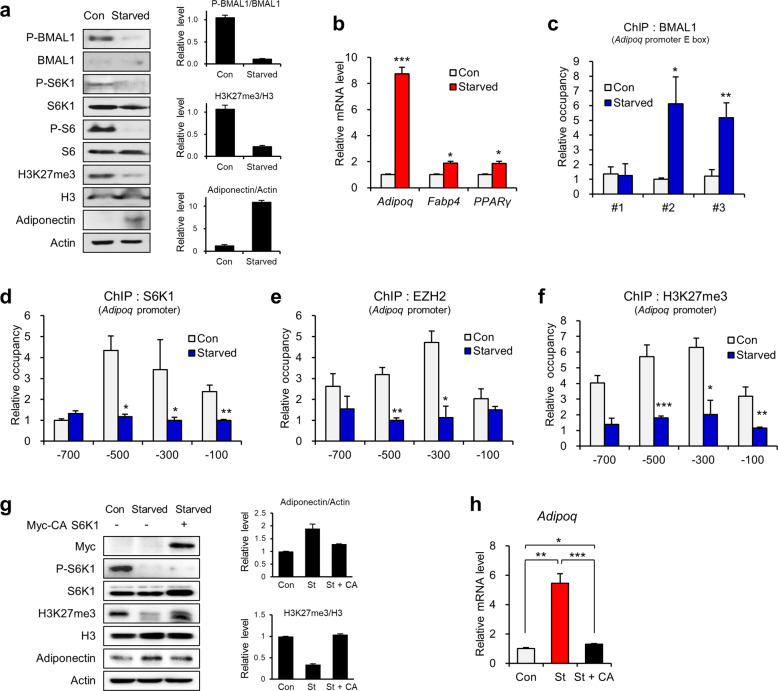
Starvation induces EZH2-to-BMAL1 transcriptional switching to promote adiponectin expression. **a** Immunoblot analysis of the 3T3-L1 adipocytes incubated with serum-starved medium for 6 h. **b** The mRNA levels of *Adipoq*, *Fabp4*, and *Pparγ* in the 3T3-L1 adipocytes incubated with serum-starved medium for 6 h. **c** ChIP-qPCR analysis using BMAL1 antibody on E-box #1, #2, and #3 of *Adipoq* in the 3T3-L1 adipocytes incubated with serum-starved medium for 6 h. **d-f** ChIP-qPCR analysis using S6K1 (**d**), EZH2 (**e**), and H3K27me3 (**f**) antibodies of the promoter regions (−700, −500, −300, and −100 bp) of *Adipoq* in the 3T3-L1 adipocytes incubated with serum-starved medium for 6 h. **g**, **h** Immunoblot analysis (**g**) and mRNA level (**h**) of the 3T3-L1 adipocytes overexpressing CA-S6K1 incubated with serum-starved medium for 6 h. Data are represented as the mean ± SEM for *n* = 3. *P < 0.05; **P < 0.01; ***P < 0.001.

Next, we examined S6K1-mediated transcriptional switching between BMAL1 and the H2BS36p/EZH2/H3K27me3 axis under nutrient-controlled conditions. In the white adipose tissue of fasted mice, adiponectin expression was upregulated, but phospho-S6K1, phospho-BMAL1, and H3K27me3 levels were decreased (Supplementary Fig. 3c, d). Consistent with this finding, downregulated levels of phospho-BMAL1 Ser42, phospho-S6K1 Thr389 and H3K27me3 were observed in 3T3-L1 adipocytes upon starvation, whereas both protein and mRNA levels of adiponectin were increased (Fig. 5a, b). The increased expression of adiponectin was caused by enhanced recruitment of BMAL1 to the #2 and #3 E-box regions of *Adipoq* upon starvation (Fig. 5c) and diminished enrichment of S6K1, EZH2, and H3K27me3 (Fig. 5d–f). However, ectopic expression of constitutively active (CA) S6K1 restored the starvation-mediated effects, such as reduced H3K27me3 and increased adiponectin expression, resulting in similar levels of H3K27me3 and adiponectin to those of the control group (Fig. 5g, h). Next, we assessed the phosphorylation level of BMAL1 under nutrient-rich conditions using mice with high-fat diet (HFD)-mediated obesity. Consistent with our previous observation of the hyperactive S6K1-H2BS36p-H3K27me3 axis in obese adipose tissues^9^, phospho-BMAL1 Ser42 levels were also elevated in epididymal adipose tissues from the obese mice (Supplementary Fig. 3e). Collectively, the data indicate that phosphorylation of BMAL1 by S6K1 in the nucleus leads to the dissociation of BMAL1 from the *Adipoq* promoter, which is required for the recruitment of the S6K1/H2BS36p/H3K27me3 axis to suppress adiponectin expression.

### S6K1 induces switching of transcriptional modulators to suppress adiponectin expression

**Fig. 6:**
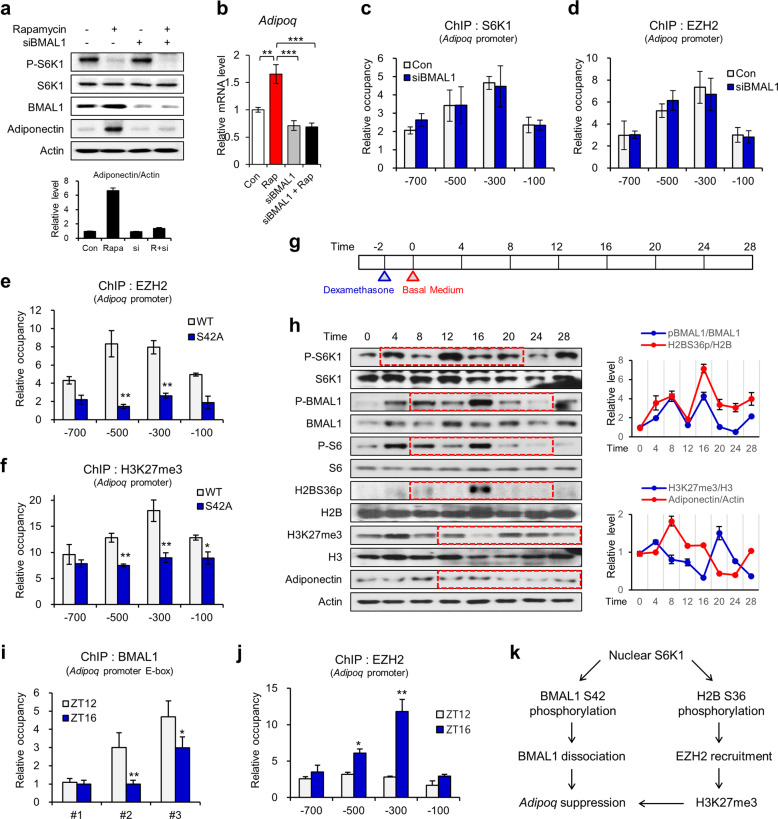
S6K1 induces BMAL1-to-EZH2 transcriptional switching to suppress adiponectin expression. **a** Immunoblot analysis of the 3T3-L1 adipocytes transfected with BMAL1 siRNA and treated with rapamycin (25 nM) for 3 h. **b** The mRNA levels of *Adipoq* in the 3T3-L1 adipocytes transfected with BMAL1 siRNA and treated with rapamycin (25 nM) for 3 h. **c**, **d** ChIP-qPCR analysis using S6K1 (**c**) and EZH2 (**d**) antibodies on the promoter regions (−700, −500, −300, and −100 bp) of *Adipoq* in the 3T3-L1 adipocytes transfected with BMAL1 siRNA. **e**, **f** ChIP-qPCR analysis using EZH2 (**e**) and H3K27me3 (**f**) antibodies of the promoter regions (−700, −500, −300, and −100 bp) of *Adipoq* in the 3T3-L1 adipocytes overexpressing BMAL1-WT or BMAL1-S42A. **g** Schematic representation of the experiment performed to evaluate the circadian rhythmic expression of samples. The 3T3-L1 adipocytes were synchronized for 2 h using dexamethasone followed by harvesting at 4 h intervals for 28 h. **h** Immunoblot analysis of the 3T3-L1 adipocytes harvested at the indicated time points after synchronization. The protein levels were quantified by ImageJ. The graphs represent the levels of the phosphorylated form normalized to those of the total form. **i** ChIP-qPCR analysis using the BMAL1 antibody of E-box #1, #2, and #3 of *Adipoq* in the 3T3-L1 adipocytes at ZT12 and ZT16. **j** ChIP-qPCR analysis using EZH2 antibody of the promoter regions (−700, −500, −300, and −100 bp) of *Adipoq* in the 3T3-L1 adipocytes at ZT12 and ZT16. **k** Schematic pathway demonstrating how S6K1 suppresses *Adipoq* expression through the H2BS36p/EZH2/H3K27me3 axis and BMAL1 phosphorylation. Data are represented as the mean ± SEM for *n* = 3. *P < 0.05; **P < 0.01; ***P < 0.001.

Next, we investigated whether BMAL1 is involved in S6K1/EZH2-mediated control of adiponectin expression. Upon the depletion of BMAL1, adiponectin expression was constitutively repressed even when adipocytes were treated with rapamycin, although the adiponectin level was highly elevated by rapamycin in the presence of BMAL1 (Fig. 6a, b). However, BMAL1 knockdown alone did not change the adiponectin levels compared to those of the control group without rapamycin (Fig. 6a, b). Consistently, recruitment of both S6K1 and EZH2 to the adiponectin promoter was not significantly altered by BMAL1 depletion (Fig. 6c, d). These data indicate that BMAL1 does not contribute to adiponectin expression under basal conditions when S6K1 is activated, but BMAL1 is involved in the elevation of adiponectin transcription upon S6K1 inhibition. To identify the regulatory mechanism of EZH2/H3K27me3 in BMAL1 transcriptional activity, we performed ChIP-qPCR analysis with adipocytes expressing the phospho-resistant BMAL1-S42A mutant. The enrichment of EZH2 and H3K27me3 on the *Adipoq* promoter was reduced by BMAL1-S42A compared to that by BMAL1 WT (Fig. 6e, f). These results suggest that the epigenetic repression of adiponectin through EZH2/H3K27me3 disappears upon BMAL1-mediated transcription.

A previous study reported the circadian-rhythmic expression patterns of adiponectin, which is regulated by CLOCK:BMAL1 transcription factors in adipocytes^12^. To investigate whether S6K1-mediated phosphorylation of BMAL1 is essential for the circadian rhythmic expression of adiponectin, we induced circadian synchronization of fully differentiated adipocytes using dexamethasone^13^ and collected samples every four zeitgeber times (ZT; time post-synchronization) from ZT0 to ZT28 (Fig. 6g). The phospho-BMAL1 and H2BS36p levels showed similar circadian-rhythmic patterns, whereas the levels of H3K27me3 and adiponectin showed opposite trends (Fig. 6h). The adiponectin expression level was highest in ZT8, followed by a decrease during ZT8-ZT24, and then recovered to basal levels at ZT28 (Fig. 6h). However, in the adipocytes treated with rapamycin during circadian synchronization and release, adiponectin levels drastically decreased during ZT8-ZT12 and then rapidly recovered from ZT12 for a peak at ZT24 (Supplementary Fig. 4a). These results suggest that coordinated epigenetic changes mediated by S6K1 in adipocytes are required for the circadian-rhythmic expression of adiponectin along with BMAL1 transcriptional activity.

A previous report demonstrated that cytoplasmic BMAL1 decreases from ZT16 until ZT28^6^. Our data also showed that cytosolic BMAL1 was markedly increased at ZT16 compared to that at ZT12 (Supplementary Fig. 4b), indicating a potential functional change in BMAL1 in distinct subcellular compartments between ZT12 and ZT16. We also found occupancy switching between BMAL1 and EZH2 at ZT12 and ZT16, times at which adiponectin expression was downregulated. The enrichment of BMAL1 on the #2 and #3 E-box regions of the *Adipoq* promoter was decreased, whereas the binding of EZH2 was increased at ZT16 compared to ZT12 (Fig. 6i, j). These data indicate that the transcriptional switching mechanism between BMAL1 and EZH2 is essential for the precise regulation of adiponectin expression in a circadian manner.

## Discussion

Adiponectin secreted from white adipose tissue is inversely associated with many risk factors involved in metabolic disorders that regulate glucose and lipid metabolism and insulin sensitivity^14^. Thus, increasing adiponectin production is considered a useful strategy for the treatment of metabolic diseases. Here, we focused on the regulatory role of S6K1 in adiponectin expression. We suggest dual pathways in which S6K1 finely controls adiponectin expression upon diverse environmental cues (Fig. 6k). S6K1 activates the H2BS36p/EZH2/H3K27me3 axis, which represses adiponectin expression. In parallel, BMAL1, which is phosphorylated by S6K1, dissociates from the *Adipoq* promoter to induce EZH2 recruitment, which leads to suppression of adiponectin expression in a circadian-rhythmic manner. In contrast to the finding of this molecular cascade, earlier studies have shown that plasma adiponectin levels were reduced in S6K1 KO mice^15,16^. This inconsistency apparently results from the complicated systemic action of S6K1, given that hypothalamic S6K1 exhibits protective effects against a high-fat diet, which are antagonistic effects of peripheral S6K1^17^. Another possible reason for the decreased total plasma level of adiponectin is the extreme decrease in body fat mass of the S6K1 KO mice because adiponectin is mainly produced by adipocytes^15,18^. For these reasons, long-term depletion of S6K1 is not suitable to investigate the molecular link between S6K1 and adiponectin. Here, we focused on the effects of acute inhibition of S6K1 by rapamycin or starvation on adiponectin expression in adipocytes.

Although an earlier study reported that mTOR/S6K1 signaling regulates the circadian clock by affecting BMAL1, the researchers demonstrated only translational regulation in the cytoplasm but did not identify transcriptional alterations by S6K1-mediated circadian clock regulation^6^. Our present findings add accurate molecular links in which S6K1 directly affects epigenetic regulation of adiponectin in the nucleus. We observed that S6K1 interacts with BMAL1, inducing its phosphorylation in both the cytoplasm and nucleus (Fig. 4a, b). In addition, BMAL1 dissociated from chromatin encoding the *Adipoq* promoter and diffused to the cytoplasm between 12 and 16 h after circadian synchronization (Fig. 6i and Supplementary Fig. 4b). Collectively, the functions of BMAL1 in two different subcellular compartments are controlled in a distinct but timely manner by S6K1 activity.

Conventionally, numerous studies on the biological role of S6K1 have mainly focused on its translational regulation in the cytoplasm. However, our current study found that S6K1 and H3K27me3 are segregated as puncta in the nucleus and diffuse upon adipogenesis, which shows the nuclear rearrangements of S6K1-mediated H3K27me3 according to cell status. Our previous research showed that S6K1-mediated H2BS36p is required for EZH2-mediated H3K27me3 on *Wnt* genes, leading to their suppression during early adipogenesis. This current study revealed that the epigenetic function of S6K1 is not limited to early adipogenesis but also applies to fully differentiated adipocytes that regulate the expression of an important adipokine, adiponectin. Given that excessive adipogenesis is observed only in childhood obesity, our finding demonstrating the role of S6K1 in fully differentiated adipocytes will provide important insights to help understand the pathophysiology of adult obesity and obesity-related metabolic disorders.

The expression level of adiponectin peaks in the morning, which is related to fatty acid withdrawal and glucose tolerance improvement^19^. While PPARγ is considered a key regulator of the circadian rhythmic expression of adiponectin^20^, our data showed that adiponectin expression was upregulated in fasted mice, even though PPARγ expression was downregulated (Supplementary Fig. 3). This result suggests that PPARγ is not the only factor that controls the circadian rhythmic expression of adiponectin. In this study, we demonstrated that BMAL1 could specifically modulate the transcription of *Adipoq* by directly binding to the E-box of the *Adipoq* promoter. The transcriptional regulation of adiponectin is dependent on the crosstalk between the dynamic changes in BMAL1 enrichment and the S6K1/H2BS36p/H3K27me3 axis on the *Adipoq* promoter. In particular, our data demonstrated that phosphorylation of BMAL1 by S6K1 resulted in dissociation of BMAL1 from the *Adipoq* promoter, thereby allowing the binding of EZH2 to the promoter region.

The daily expression of adiponectin is also affected by physiological nutrient status^21,22^. Both a high-fat diet and fasting disrupt the circadian fluctuation of adiponectin signaling by inducing delay or advance of the phase, respectively^21,22^. Likewise, we observed that S6K1 inhibition accelerated the phase involving upregulation of adiponectin expression, which is similar to fasting-mediated effects (Supplementary Fig. 4a). We also showed coupled alterations in S6K1 activity, phosphorylated BMAL1, and adiponectin suppression upon fasting. According to previous literature, the metabolic actions of adiponectin share many similarities with key features of the S6K1 knockout mice. Our current data accurately demonstrate the molecular mechanisms underlying these physiological alignments.

In conclusion, we unraveled a novel epigenetic switch system between EZH2 and BMAL1 by which S6K1 controls the production of adiponectin, a fat-derived hormone that displays beneficial effects on metabolism. These findings suggest multidirectional functions of S6K1 in the transcriptional regulation of adiponectin and thus provide a better understanding of the molecular mechanism underlying the rhythmic expression of adiponectin.

## Supplementary information

Supplementary Materials
