# Supplementary material for: S6K1 controls adiponectin expression by inducing a transcriptional switch: BMAL1-to-EZH2
Source: Exp Mol Med. 2022 Mar 25;54(3):324–33. doi: 10.1038/s12276-022-00747-7 (PMC8979988; doi:10.1038/s12276-022-00747-7)
Supplement: Supplementary file 1 — Supplementary Materials [file 12276_2022_747_MOESM1_ESM.pdf]

Supplementary Materials for

S6K1 Controls Adiponectin Expression by Inducing A Transcriptional Switch: BMAL1-to-EZH2

Sang Ah Yi*, Ye Ji Jeon, Min Gyu Lee, Ki Hong Nam, Sora Ann, Jaecheol Lee*, Jeung-Whan Han*

*Corresponding author. Email: angelna1023@hanmail.net (S.A.Y.), jaecheol@skku.edu (J.L.), jhhan551@skku.edu (J.W.H.),

This file includes:

Supplementary Fig. 1. Rearrangement of S6K1 and EZH2 in mature adipocytes

Supplementary Fig. 2. Negative correlation between adiponectin and S6K1/EZH2 axis

Supplementary Fig. 3. S6K1-dependent BMAL1 phosphorylation downregulates *Adipoq* expression

Supplementary Fig. 4. Phosphorylation and subcellular localization of BMAL1 after circadian synchronization.

Supplementary Table 1. Antibodies used in this study

Supplementary Table 2. Primers used in this study

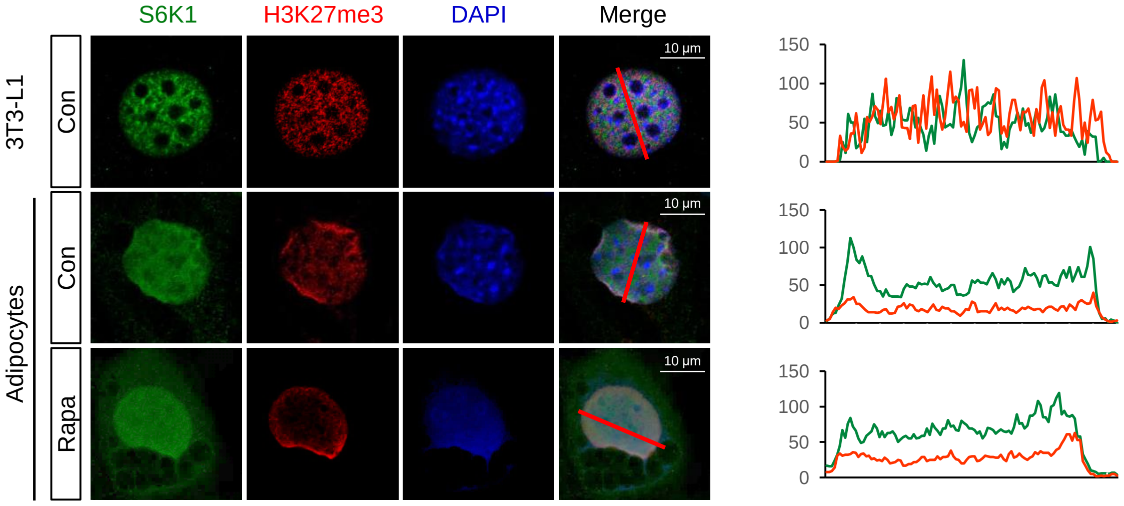

Supplementary Fig. 1 Rearrangement of S6K1 and EZH2 in mature adipocytes. Immunocytochemistry analysis of 3T3-L1 preadipocytes and fully differentiated 3T3-L1 adipocytes. S6K1 (green, first lane) and H3K27me3 (red, second lane) merged with the nuclear stain DAPI (blue, third lane).

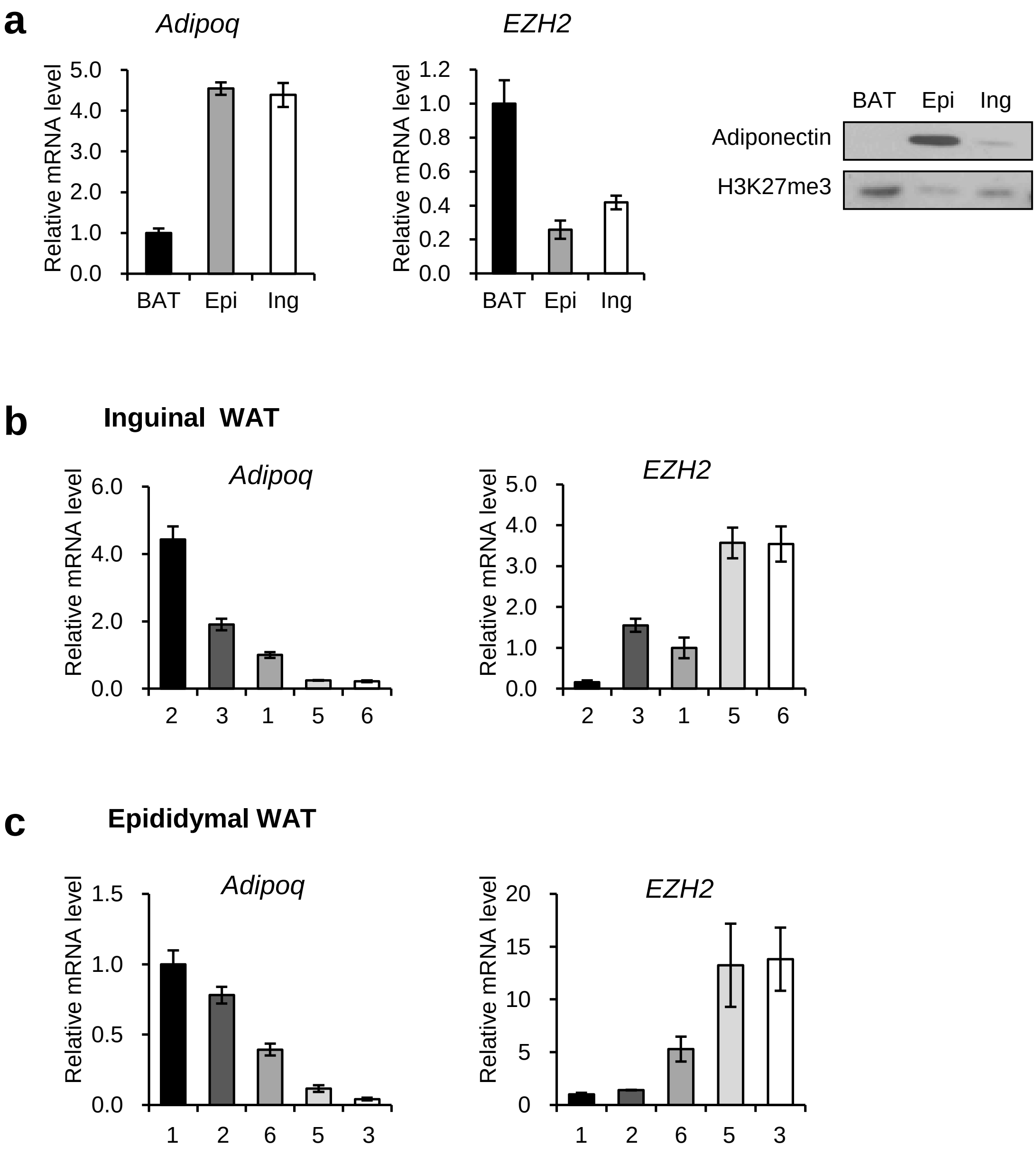

Supplementary Fig. 2 Negative correlation between adiponectin and S6K1/EZH2 axis.
(**a**) The mRNA levels of *Adipoq* and *Ezh2* in brown adipose tissue, epididymal white adipose tissue (Epi), and inguinal white adipose tissue (Ing) extracted from CF-1 mice.
(**b**) The mRNA levels of *Adipoq* and *Ezh2* in inguinal white adipose tissue of five CF-1 mice.
(**c**) The mRNA levels of *Adipoq* and *Ezh2* in epididymal white adipose tissue of five CF-1 mice.

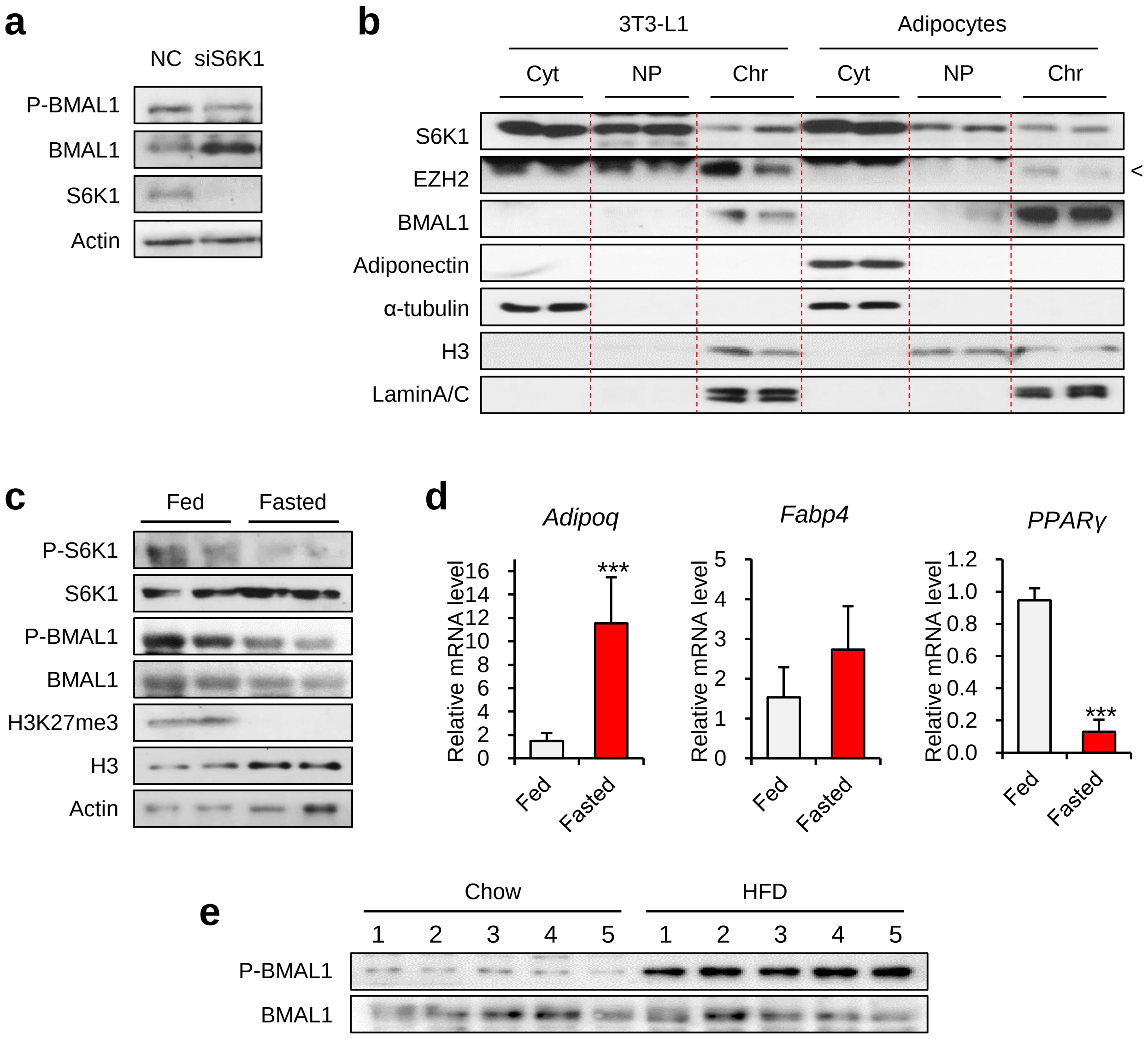

Supplementary Fig. 3 S6K1-dependent BMAL1 phosphorylation downregulates *Adipoq* expression.

(**a**) Immunoblot analysis of 3T3-L1 adipocytes expressing S6K1 siRNA (#03) or negative control siRNA.

(**b**) Immunoblot analysis of cytoplasm (Cyt), nucleoplasm (NP), and chromatin (Chr) fractions isolated from 3T3-L1 preadipocytes and fully differentiated 3T3-L1 adipocytes.

(**c**) Immunoblot analysis of epididymal white adipose tissues obtained from fed or fasted (24 h) mice.

(**d**) The mRNA levels of *Adipoq*, *Fabp4*, and *Ppar*γ in epididymal white adipose tissues obtained from fed or fasted (24 h) mice.

(**e**) Immunoblot analysis of epididymal white adipose tissues obtained from mice fed with normal chow diet (chow) or high-fat diet (HFD) for 10 weeks.

Data are represented as the mean ± SEM for n=3. *P < 0.05; **P < 0.01; ***P < 0.001.

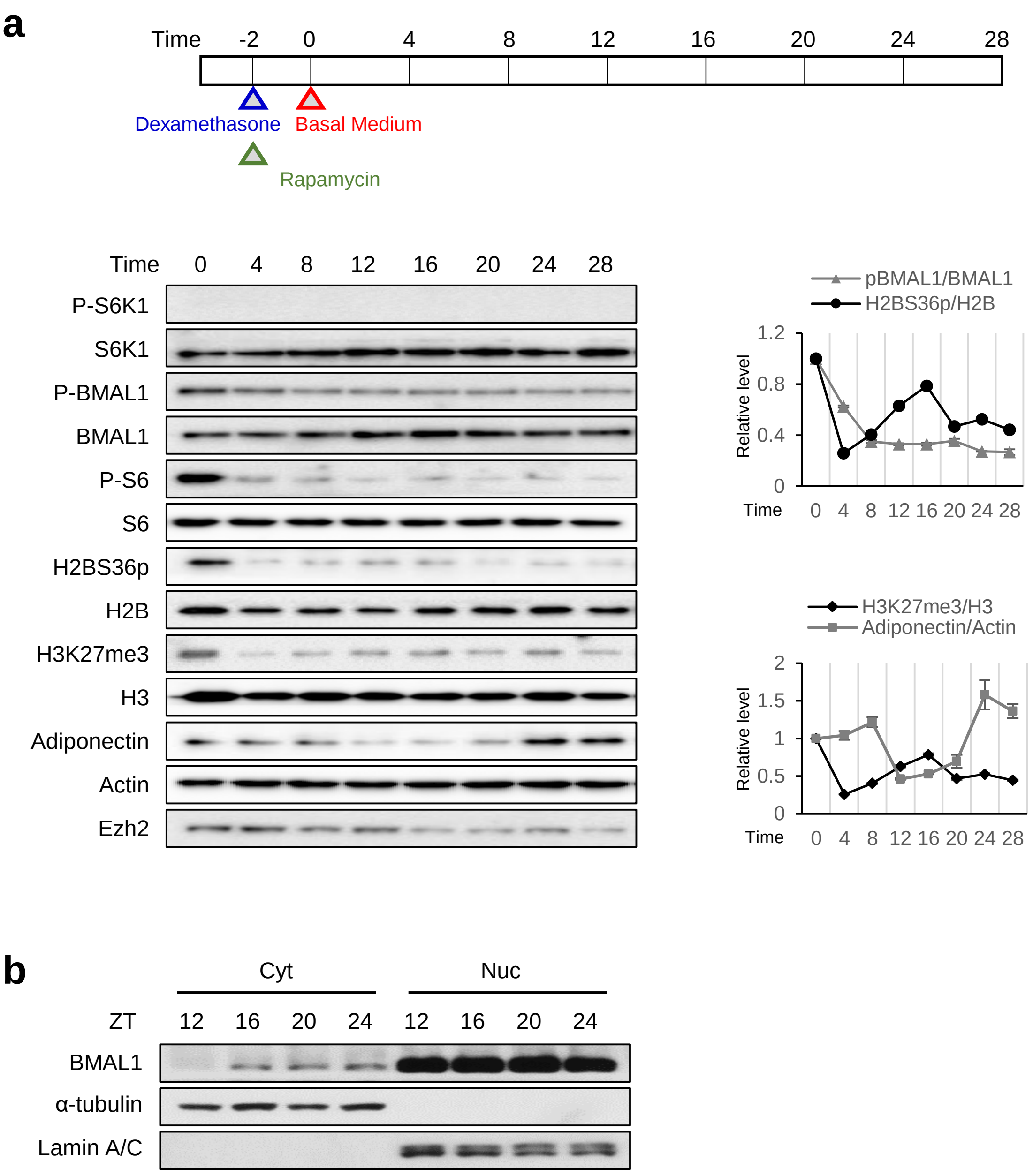

Supplementary Fig. 4 Phosphorylation and subcellular localization of BMAL1 after circadian synchronization.

(**a**) Immunoblot analysis of rapamycin (25 nM)-treated 3T3-L1 adipocytes harvested at indicated time points after synchronization. The protein levels were quantified by Image J.

(**b**) Immunoblot analysis of separated cytosolic and nuclear extracts from 3T3-L1 adipocytes at ZT12, ZT16, ZT20, and ZT24.

Supplementary Table 1. Antibodies used in this study

Antibodies	Application	Source	Catalog number
Anti-Actin	Immunoblotting	Merck/Millipore	MAB1501
Anti-Adiponectin	Immunoblotting	Cell signaling technology	2789
Anti-α-tubulin	Immunoblotting	SantaCruz	sc-32293
Anti-BMAL1	Immunoblotting, chromatin immunoprecipitation	SantaCruz	sc-365645
Anti-phospho-BMAL1 (Ser42)	Immunoblotting	Cell signaling technology	13936
Anti-EZH2	Immunoblotting, chromatin immunoprecipitation	Cell signaling technology	5246
Anti-Flag	Immunoblotting, chromatin immunoprecipitation	Sigma	F1804
Anti-H2BS36p	Immunoblotting, chromatin immunoprecipitation	ECM Bio Science	HP4331
Anti-H3K27me3	Immunoblotting, chromatin immunoprecipitation, immunofluorescence	Millipore	07-449
Anti-Histone H2B	Immunoblotting	Millipore	07-371
Anti-Histone H3	Immunoblotting	Abcam	ab1791
Anti-Lamin A/C	Immunoblotting	Cell signaling technology	2032
Anti-S6	Immunoblotting	Cell signaling technology	2217
Anti-phospho-S6 (Ser235/236)	Immunoblotting	Cell signaling technology	4858
Anti-S6K1	Immunoblotting	Cell signaling technology	9202
Anti-S6K1	Immunoprecipitation, chromatin immunoprecipitation, immunofluorescence	SantaCruz	sc-8418
Anti-phospho-S6K1 (Thr389)	Immunoblotting	Cell signaling technology	9205

Supplementary Table 2. Primers used in this study

Primer	Application	Sequence (5'-3')
S6k1 Forward	RT-qPCR	ATATGAACTTGGCATGGAAC
S6k1 Reverse	RT-qPCR	TTGCTCCTGTTACTTTTCGT
Adipoq Forward	RT-qPCR	TGTTCCTCTTAATCCTGCCCA
Adipoq Reverse	RT-qPCR	CCAACCTGCACAAGTTCCCTT
Pparγ Forward	RT-qPCR	GCATGGTGCCTTCGCTGA
Pparγ Reverse	RT-qPCR	TGGCATCTCTGTGTCAACCATG
β-actin Forward	RT-qPCR	ACGGCCAGGTCATCACTATTG
β-actin Reverse	RT-qPCR	TGGATGCCACAGGATTCCA
Adipsin Forward	RT-qPCR	CATGCTCGGCCCTACATG
Adipsin Reverse	RT-qPCR	CACAGAGTCGTCATCCGTCAC
Fabp4 Forward	RT-qPCR	AAGGTGAAGAGCATCATAACCCT
Fabp4 Reverse	RT-qPCR	TCACGCCTTTCATAACACATTCC
Ezh2 Forward	RT-qPCR	TGGACCACAGTGTTACCAGCA
Ezh2 Reverse	RT-qPCR	TGGGCGTTTAGGTGGTGTCT
700 bp upstream promoter of *Adipoq* gene Forward	ChIP-qPCR	ACCCCTGAACTTGCTTCACACC
700 bp upstream promoter of *Adipoq* gene Reverse	ChIP-qPCR	TGCTAGACCGGATCCCATACTGA
500 bp upstream promoter of *Adipoq* gene Forward	ChIP-qPCR	TGCATGCATATTTGCACACCAA
500 bp upstream promoter of *Adipoq* gene Reverse	ChIP-qPCR	TCAATTCCCAGCACCCACAGTA
300 bp upstream promoter of *Adipoq* gene Forward	ChIP-qPCR	ATGGCTGAACCACACAGCTTCA
300 bp upstream promoter of *Adipoq* gene Reverse	ChIP-qPCR	AGGGGTCAGGAGACCTCCCTTT
100 bp upstream promoter of *Adipoq* gene Forward	ChIP-qPCR	TTCCCAGACCCAAGCTGGATTA
100 bp upstream promoter of *Adipoq* gene Reverse	ChIP-qPCR	CCACCCAGTCAAGGCCAATAGC
E-box #1 in promoter of *Adipoq* gene Forward	ChIP-qPCR	GTATGGGATCCGGTCTAGCA
E-box #1 in promoter of *Adipoq* gene Reverse	ChIP-qPCR	AATGACACCCACGACCCTTA
E-box #2 in promoter of *Adipoq* gene Forward	ChIP-qPCR	GCCCCTCTTCATTCTTACTG
E-box #2 in promoter of *Adipoq* gene Reverse	ChIP-qPCR	TAGCCTTTCCCTCCAGAGGA
E-box #3 in promoter of *Adipoq* gene Forward	ChIP-qPCR	AGAAGCTCTACTTGGCTTCCC
E-box #3 in promoter of *Adipoq* gene Reverse	ChIP-qPCR	ACCATTCGACCCCAGACG
